# Development of Antiatherosclerotic Drugs on the basis of Natural Products Using Cell Model Approach

**DOI:** 10.1155/2015/463797

**Published:** 2015-06-09

**Authors:** Alexander N. Orekhov, Igor A. Sobenin, Victor V. Revin, Yuri V. Bobryshev

**Affiliations:** ^1^Department of Biophysics, Biological Faculty, Moscow State University, Moscow 119991, Russia; ^2^Institute of General Pathology and Pathophysiology, Moscow 125315, Russia; ^3^Russian Cardiology Research and Production Center, Moscow 121552, Russia; ^4^Biological Faculty, N.P. Ogaryov Mordovian State University, Republic of Mordovia, Saransk 430005, Russia; ^5^Institute for Atherosclerosis Research, Skolkovo Innovation Center, Moscow 121609, Russia; ^6^Faculty of Medicine, School of Medical Sciences, University of New South Wales, Sydney, NSW, Australia; ^7^School of Medicine, University of Western Sydney, Campbelltown, NSW, Australia

## Abstract

Atherosclerosis including its subclinical form is one of the key medical and social problems. At present, there is no therapy available for widespread use against subclinical atherosclerosis. The use of synthetic drugs for the prevention of arteriosclerosis in its early stages is not sufficient because of the limited indications for severe side effects and high cost of treatment. Obviously, effective antiatherosclerotic drugs based on natural products would be a preferred alternative. Simple cell-based models for testing different natural products have been developed and the ability of natural products to prevent intracellular lipid accumulation in primary cell culture was evaluated. This approach utilizing cell models allowed to test effects of such direct antiatherosclerotic therapy, analyzing the effects mimicking those which can occur “at the level” of arterial wall via the inhibition of intracellular lipid deposition. The data from the carried out clinical trials support a point of view that the identification of antiatherosclerotic activity of natural products might offer a great opportunity for the prevention and treatment of atherosclerotic disease, reducing cardiovascular morbidity and mortality.

## 1. Introduction

Atherosclerosis is one of the key medical and social problems, since its clinical manifestations take a leading place in “the structure” of overall morbidity and mortality [1]. Atherosclerosis is a multifactorial disease, which is characterized, in general, by the development of degenerative changes in the wall of large arteries, followed by occlusion of the lumen and that limits blood supply to organs and tissues. Subclinical (asymptomatic) atherosclerosis is the most widespread pathology. It is well known that atherosclerotic lesions exist already in young people, steadily progressing over decades until clinical manifestations occur [1–4]. In the middle age, individuals are usually free from clinical manifestations of atherosclerosis while, as a matter of fact, the incidence of atherosclerotic lesions accounts for nearly 100% [2–4].

At present, there are no methods of “direct” antiatherosclerotic prevention and therapy available for widespread use in subclinical atherosclerosis. The main reason for this is the still existing uncertainty about the exact mechanisms of human atherogenesis and, as a result, about the most appropriate therapeutic targets. In epidemiological studies, a number of factors, associated with the increased risk of vascular occlusion, have been identified [5]; those conventional risk factors for atherosclerosis development include several clinical and biochemical syndromes, which contribute to the development of pathology. It is not surprising, therefore, that the elimination of risk factors is the most extensively studied area and is a widely used approach for the primary prevention of atherosclerosis [6]. However, this approach provides just an indirect impact, since it is directed to the changing of several conditions which are not “immediately” and directly related to the molecular and cellular mechanisms of atherogenesis but are hindering the emergence and progression of atherosclerotic lesions.

On the opposite side, an idea of a so called “direct” antiatherosclerotic impact of therapy does exist and is thought to be a pathogenetic approach to prevent the onset and progression of atherosclerotic lesions by inhibiting the molecular and cellular mechanisms of atherogenesis thus causing prevention or regression of atherosclerosis [7]. Currently, such approach to pathogenetic atherosclerosis prevention and treatment is under development.

It should be noted here that a wide use of synthetic drugs for the prevention of arteriosclerosis in the early stages of the disease development may be impractical, because of the limited indications for use, severe side effects, and high cost of treatment. Currently, discussion about drugs possessing antiatherosclerotic action focuses primarily on statins [8]. However, it is known that regular long-term statin therapy leads to a cessation of development and regression of existing atherosclerotic lesions. It has become clear that the widespread use of statins for the prevention of atherosclerosis in its early stages seems to be unlikely because of the narrow indications for prescription and severity of side effects. Effective antiatherosclerotic drugs based on natural products can be a preferred alternative [9]. For early prevention of atherosclerosis, nonpharmaceutical medicines of natural origin may be suggested, as they have virtually no side effects, may have physiological regulatory effect, and, as a consequence, may allow for a lifetime appointment. The remedies of natural origin may have a wider range of effects than drugs, affecting a number of risk factors for atherosclerosis, thus possessing not only a direct antiatherosclerotic action at the cellular level, but also indirect effects (e.g., cholesterol lowering and blood pressure regulation). The basic principle for the use of drugs and nonpharmaceutical remedies for the prevention of atherosclerosis should be the pathogenetic mechanism of action and the effectiveness confirmed in clinical studies.

Using natural products for the prevention of atherosclerosis should be based on their ability to prevent the accumulation of cholesterol in the cells of the arterial wall that could be prevented at the initial stage of atherogenesis at cellular level. Currently, parapharmaceuticals based on botanicals and other natural products are increasingly attracting researcher interest. Nevertheless, understanding their possible cardioprotective effects and limited appreciation of their possible impact on risk factors for cardiovascular disease such as lowering of blood cholesterol and blood pressure regulation [7, 10–12] are still limited. It is clear that the development of a new methodology for assessing the therapeutic potential of natural products would be obviously beneficial. Until recently there were no methodological approaches to the assessment of antiatherosclerotic potential of natural substances. One of the main obstacles for the development of pathogenetic therapy of atherosclerosis, especially against the early subclinical stages of the disease development, is the lack of adequate pathophysiological models and the absence of a proper algorithm for the development of drug and parapharmaceuticals that would possess direct antiatherosclerotic action.

In this review, we highlight achievements in the development and use of models based on primary cultures of human aortic cells which were already utilized for search of antiatherosclerotic agents of natural origin. In order to explain the peculiarities and usefulness of such models for testing properties of natural substances against atherosclerotic process, we firstly here briefly describe some key atherosclerotic mechanisms which can and should be targeted.

## 2. Mechanisms of Human Atherogenesis

Current understanding of cellular and molecular mechanisms of atherogenesis is based on the classical lipid theory of atherosclerosis, postulating that the most important event in the development of atherosclerosis is the accumulation of extracellular and intracellular lipids in the arterial intima [13, 14]. The major source of lipids accumulating in intimal cells is low-density lipoprotein (LDL). It should be stressed here that chemical modification of lipoprotein particles is evidently necessary for the manifestation of atherogenic effect, since native (intact) LDL does not cause the accumulation of lipids in cells populating the arterial wall. The range of exactly known atherogenic LDL modifications is limited to three options: desialylation, the change of the total surface charge, and the change of hydrated density of lipoprotein particles; all of them may be accompanied by oxidation [15–18]. In fact, in all cases we actually deal with the same type of multiple atherogenic modifications, but differently evaluated by different methods of laboratory diagnostics [15]. If there is a sufficient amount of modified LDL in the circulation, the additional mechanisms enhancing LDL atherogenic potential come into action. The main one is the formation of large lipid-containing complexes. It is known that modified LDL due to the changes in surface charge acquires the ability to spontaneous self-association. Additionally, modified LDL possesses antigenic properties, thus inducing the production of anti-apoB autoantibodies, which ultimately leads to the formation of LDL-containing circulating immune complexes. Modified LDL also has high avidity for connective tissue matrix components. All the above mentioned processes lead to the appearance of large LDL-containing aggregates, the metabolism of which at the cellular level is operated by alternative pathways other than the classical receptor pathway. The main mechanism of internalization of such particles by vascular cells is uncontrolled phagocytosis. The pathways of intracellular degradation of LDL-containing phagocytized particles differ significantly from the classic native LDL metabolism; therefore, it occurs in “a short time” and leads to massive intracellular accumulation of the residues of lipoprotein particles, mainly in the form of lipid droplets. Under histological examination, these lipid-laden cells are defined as foam cells, the presence of which is an essential feature and key attribute of atherosclerotic lesions.

**Figure 1:**
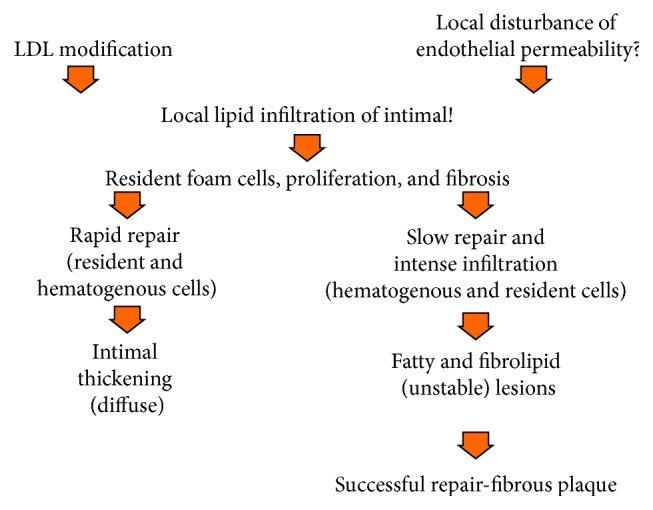
Scheme showing an association of consecutive events relating to the initiation and development of atherosclerotic lesions.

A scheme, presented in Figure 1, is based on findings produced during studies of human atherosclerosis; this scheme incorporates the key events of atherogenesis (Figure 1). Such a scheme not only explains the accumulated facts about the development of atherosclerosis but also might suggest how to develop novel approaches for the prevention and treatment of atherosclerosis. For the development of initial focal lesion in arterial wall, at least two events must cooccur, namely, atherogenic modification of LDL circulating in the blood and the local change in endothelial permeability. It is important to mention that modification of LDL can occur also directly in the intima, after penetration of LDL via the luminal endothelium. The causes of LDL modification are the processes occurring in both the blood and the vascular wall leading to multiple changes in the physical and chemical characteristics of lipoprotein and consequently to a disturbance of the functions. One of the earliest events of multiple modifications of LDL is a desialylation of lipoprotein glycoconjugates caused by the transsialidase circulating in the blood [15]. Local changes in endothelial permeability might be associated with the heterogeneity of the endothelial lining, that is, the presence of clusters of multinucleated giant endothelial cells and small endothelial cells which are located mosaically [19]. Undoubtedly, different structural types of the endothelium are present along the luminal surface and differ functionally, even though the peculiarities of heterogeneity of the endothelium in human arteries are still poorly understood. One cannot exclude the possibility that mosaicism in the distribution of endothelial clusters possessing different functional properties might determine mosaicism and “focality” of the development of the atherosclerotic lesions in the arterial system. Penetration of modified LDL into the subendothelial space in loci with increased endothelial permeability leads to the formation of focal intimal lipid infiltrations. Both in the blood and in the intima modified LDLs form associates with each other or with autoantibodies against modified LDL (circulating immune complexes). Additionally, in the intima LDLs might associate with extracellular matrix components, complicating the characteristics of modified LDLs [16–18]. Modified LDLs and LDL associates interact with cells populating the subendothelial intima. Intimal cells comprise resident mesenchymal origin cells and cells that intrude the intima from the circulation (hematogenous cells). Conformation of modified LDL as a part of associate is fundamentally different from that of native lipoprotein particle, that is why LDL associates do not interact with LDL-receptors on the cell surface but rather enter the intimal cell by nonreceptor pathway. According to Goldstein and Brown [20], nonreceptor LDL uptake leads to the accumulation of intracellular lipids and foam cell formation.

Phagocytosis of LDL associates might be understood as a result of innate immunity response. An intimal cell cannot percept a LDL associate as a LDL particle, for which such cell has well-preserved mechanism of receptor interaction, internalization, and degradation. Associate, containing LDL, is likely perceived by the intimal cell as a pathogen, a subject for the destruction by phagocytosis. Phagocyte (monocyte-derived macrophage or resident pluripotent mesenchymal cell) takes up the associate, containing LDL, perceived as pathogen switching on the mechanism of innate immunity by secreting the signaling molecules which attract neighboring arterial cells and blood-origin inflammatory cells in the focus (locus) of inflammation [21]. In response to inflammatory signals, resident and inflammatory cells that accumulated in the intima also begin to take up the LDL associates and thus accumulating excess lipids and transforming into foam cells. Accumulation of lipids in arterial cells not only generates foam cells but also triggers the processes typical of reparative phase of inflammation, namely, proliferation and fibrosis (extracellular matrix synthesis); besides interaction with the LDL associates leads to a decrease in cell-to-cell contacts between resident cells [22].

With favorable developments the reparation process completes rapidly. In the areas of the intima, where such events happened, focal increase in cell number occurs, along with the increase in excessive production of extracellular matrix components. Their events occur in human arterial intima throughout life. Over time, focal fibrotic thickening, which is also characterized by an increase in cellularity, develops throughout the arterial bed and becomes diffused. Apparently, the formation of diffuse intimal thickening is typical for adult arteries.

**Figure 2:**
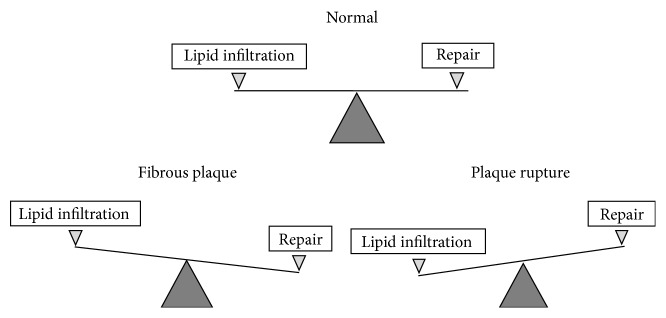
Scheme showing delicate balance between infiltrative and reparative phases in fatty atherosclerotic lesion.

However, not always the innate immune response (an inflammatory reaction) completes promptly and successfully. If, for some reasons, the local lipid infiltration of the intima does not cease immune cells will be “forced” to participate in the development of an inflammatory response, with increased intensity. Local accumulation of cells will increase and cell proliferation and fibrosis will intensify. In this scenario, reparation will proceed slowly and inefficiently, and the process would become chronic. Local chronic inflammation will be accompanied by increased lipidosis because the cells, populating this locus of intima, would not be able to effectively cope with the continuously increased lipid infiltration of the intima. Development of cellular lipidosis will thus lead to a loss of intercellular contacts, increased proliferation, and a sharp intensification of fibrosis [22]. As a result, in this area of the intima, an atherosclerotic lesion would form, initially fatty streak and eventually fibrolipid plaque. The inability of cells to effectively cope with prolonged local inflammation in the subendothelial intima will cause tissue reaction, accompanied by the formation fibrous cap in order to isolate the center of inflammation and create a barrier to prevent the further penetration of lipoproteins and immune cells from the bloodstream, which in chronic inflammatory response would contribute to and determine the pathological process. A favorable outcome in this situation would be a complete separation of the focus of inflammation with the suppression of the inflammatory response and the gradual and partial restoration of tissue functions with the formation of fibrous plaques (scar). Alternatively, unfavorable outcome with severe or even fatal consequences for the organism would be the development of fibrolipid plaque which would be prone to plaque rupture and thrombus formation [23]. Fibrolipid plaque as a stage of atherosclerotic lesion development is characterized by the occurrence of two opposing processes, namely, by infiltration and reparation (Figure 2). This equilibrium is unstable (Figure 2). If the balance is shifted toward reparation, fibrous plaque is formed which, from clinical point of view, is a favorable outcome. If reparation occurs inefficiently and lipid infiltration prevails, plaque rupture could rather occur. Such an outcome kills every second human. Thus, at both the cellular and tissue levels the most crucial event for the initiation and development of atherosclerotic lesions is lipidosis, that is, lipid infiltration of arterial intima accompanied by lipid accumulation in intimal cells. Lipidosis is a trigger for the development of atherosclerotic plaques. Appreciation of the importance of lipid accumulation in intimal cells encourages the search for approaches to prevent such event.

## 3. Cellular Models for Development of Antiatherosclerotic Drugs: Why Do We Need Such Models?

Pathogenetic approach to the prevention and treatment of atherosclerosis should be directed to search approaches that would prevent intracellular accumulation of lipids. There are at least four theoretical possibilities of how to process the intracellular accumulation of lipids; these include the following possibilities: (1) the removal of modified LDL from circulation, (2) inhibition of proatherogenic modification of native LDL, (3) the suppression of trapping of modified LDL in tissues, and (4) the removal of accumulated lipids from cells. Integrated measure of different antiatherosclerotic effects is the reduction of the rate of accumulation of intracellular lipids and the decrease of intracellular pool of cholesterol esters [24–27].

Currently, there are no drugs that would be claimed to have a direct antiatherosclerotic action. It is known that some drugs may help to reduce atherogenic potential of serum of patients with atherosclerosis [24–27]. The concept of “atherogenic potential” (“atherogenicity”) refers to the ability of the serum or its components to cause cholesterol accumulation in cells cultured from unaffected human aortic atherosclerotic intima or other types of cultured cells. The phenomenon of serum atherogenicity was first detected in patients with coronary atherosclerosis [26]. Targeted reduction of serum atherogenic potential may help prevent accumulation of lipids in cells of the arterial wall and thereby suppress atherogenesis in its initial stage [26]. Therefore, cell culture test is thought to be the most optimal and appropriate way to model the early processes of atherogenesis at the cellular level [24–27]. There is an urgent need to develop cellular models adequate for the evaluation of antiatherogenic potential of different drugs and substances. Such an approach would make it possible to perform streaming screening of drugs with potential antiatherosclerotic effect and the estimation of clinical efficacy of antiatherosclerotic therapy.

## 4. *In Vitro* Model

For the screening of antiatherosclerotic substances, a cellular model based on a primary culture of human aortic cells was developed. In this model, cells were isolated from the subendothelial part of the normal (unaffected by atherosclerosis) human aortic intima, that is, a part of the aorta which is localized between the endothelial lining and the tunica media [24–27]. For living cell isolation collagenase and elastase were used [24–26]. Using monoclonal antibodies, it has been established that cells, which were obtained from the intima and then were cultured* in vitro,* represented a mixture of different cell types [24–26], including smooth muscle cells (20–50%), pericyte-like cells (30–70%), and blood-origin cells and tissue macrophages (10%).  Table 1 shows the proportion of cell types in such primary culture. It is necessary to note here that antibodies to smooth muscle *α*-actin identify smooth muscle cells and pericytes. Antibodies 3G5 and 2A7 identify resting and active pericytes, respectively. Thus, smooth muscle cells and stellate-shaped pericyte-like cells represented the major portion of the in cell culture. Cells of hematogenous origin detectable by antibodies against leukocytes (CD45+) and tissue macrophages (CD68+) represented only a minor portion of the cultured cells.

To stimulate cellular lipidosis atherogenic serum of patients (with assessed atherosclerosis) was added to primary culture of aortic cells. Such serum has been shown to increase intracellular cholesterol approximately twofold after 24 hours of incubation in cell culture [24–26]. Together with atherogenic serum aqueous solution of substance under examination is added to the culture. If the substance reduces intracellular cholesterol accumulation caused by atherogenic serum this substance is regarded to be antiatherosclerotic agent. Antiatherosclerotic effect is expressed as percentage of suppression of intracellular cholesterol accumulation, caused by atherogenic serum.

Using this model, the effects of various drugs and chemicals were examined. Some of the substances have been found to cause antiatherosclerotic effects on cultured cells while others were ineffective in this respect; some substances have been found to possess proatherogenic effect, which is manifested in intensified accumulation of intracellular cholesterol induced by atherogenic serum (Table 2).

## 5. *Ex Vivo* Model

On the basis of primary culture of cell cultivated from the normal (unaffected by atherosclerosis) human aortic intima* ex vivo* model was also developed [24–27]. The main difference of* ex vivo* model from* in vitro* model is that not a substance but blood serum of patients after drug administration was investigated. Thereby, changes of atherogenic properties of the blood serum under the action of various preparations are evaluated. Blood is taken before and in a certain time after single dose drug administration. Sera obtained from blood samples are added to the primary culture of aortic cells.

*Ex vivo* model allows for testing not only drugs but also natural products [27]. An essential feature of the* ex vivo* model is a possibility to assess antiatherosclerotic potential of various substances and their active metabolites after digestion, distribution, and biotransformation in human organism. It is thus possible to obtain specific pharmacodynamic properties. It is known that many of the molecules present in plants are metabolized once consumed and that they are present in circulation in different chemical structures than in food. These metabolites often present different biological effects. In the case of* ex vivo* model we do not face such problems because after taking the natural product any of its metabolites are in the blood serum which we actually investigate.

**Figure 3:**
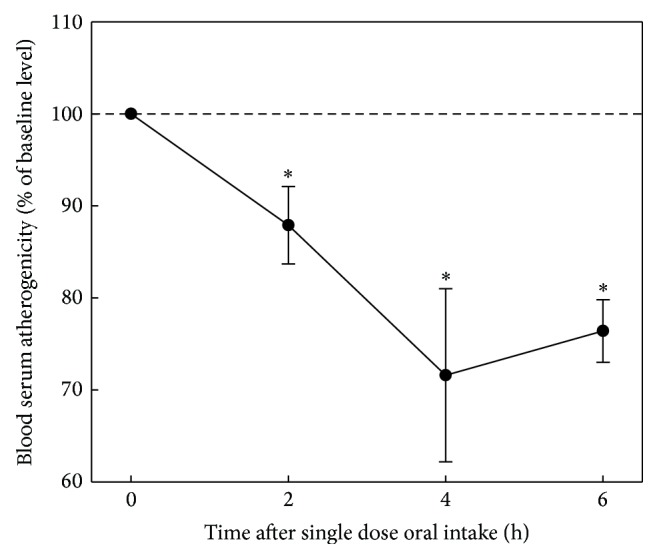
Antiatherosclerotic effect of onion in* ex vivo* model. The study involved 4 volunteers (3 males, 1 female, mean age 57 ± 5 years) whose blood serum induced 1.3–1.5-fold increase in cholesterol content of cells cultured from unaffected human aortic intima (the average level of serum atherogenicity was 141 ± 4%). Intracellular cholesterol in control cultures was 38.4 ± 1.1 mg/mg cell protein. Baseline serum atherogenicity was taken as 100%. The average values of changes of serum atherogenicity with indication of standard errors are presented.

In* ex vivo* model, a number of studies have been performed to test various natural products, mainly botanicals [27]. In screening studies volunteers (groups of 4–8 people; men and women aged 45–60 years) who had atherogenic serum were involved. The effect of a single dose of investigated natural product on atherogenicity of blood serum was evaluated. The results of the evaluation obtained in the* ex vivo* model when antiatherosclerotic effects of onion after a single dose of capsulated bulb powder (300 mg) were analyzed are presented in Figure 3. Onion powder has been found to possess antiatherogenic effect in* ex vivo* model; this effect was manifested in a moderate reduction of serum atherogenicity by 12%, 28%, and 24% from baseline after 2, 4, and 6 hours after a single dose of the preparation, respectively.

**Figure 4:**
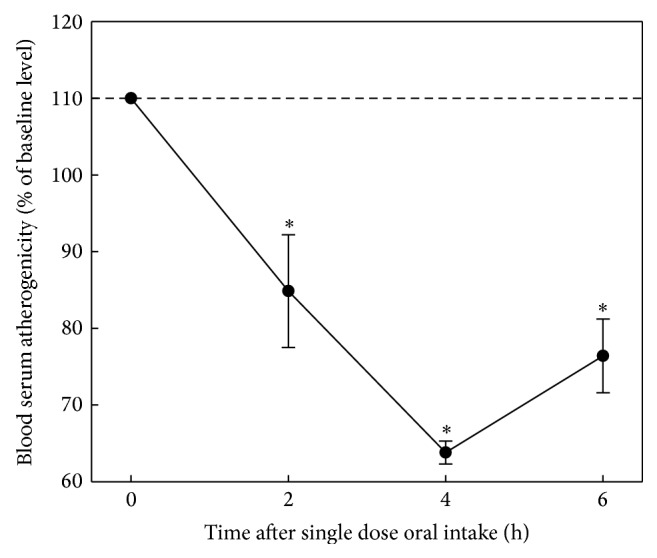
Antiatherosclerotic effect of wheat seedlings in* ex vivo* model. The study involved 8 volunteers (5 males, 3 females, mean age 51 ± 2 years) whose blood serum induced 1.7–2.3-fold increase in cholesterol content of cells cultured from unaffected human aortic intima (the average level of serum atherogenicity was 199 ± 6%). Intracellular cholesterol in control cultures was 28.0 ± 1.2 mg/mg cell protein. Baseline serum atherogenicity was taken as 100%. The average values of changes of serum atherogenicity with indication of standard errors are presented. ^∗^Significant decrease of serum atherogenicity, *p* < 0.05.

**Figure 5:**
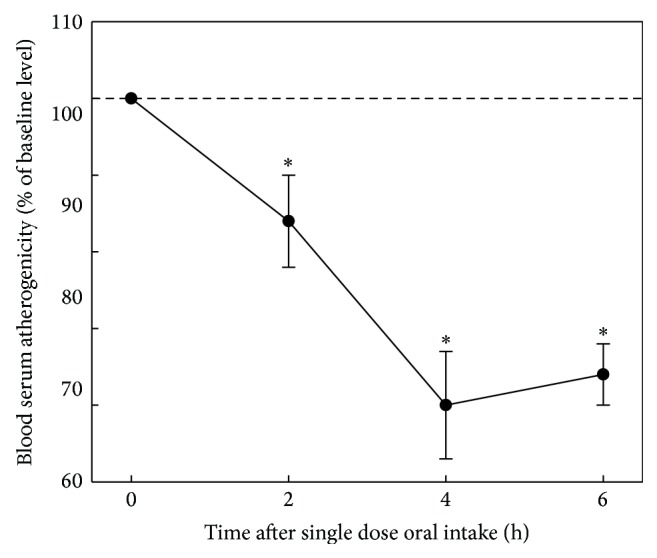
Antiatherosclerotic effect of beet juice in* ex vivo* model. The study involved 8 volunteers (6 males, 2 females, mean age 53 ± 5 years) whose blood serum induced 1.3–2.2-fold increase in cholesterol content of cells cultured from unaffected human aortic intima (the average level of serum atherogenicity was 161 ± 8%). Intracellular cholesterol in control cultures was 37.0 ± 3.6 mg/mg cell protein. Baseline serum atherogenicity was taken as 100%. The average values of changes of serum atherogenicity with indication of standard errors are presented. ^∗^Significant decrease of serum atherogenicity, *p* < 0.05.

**Figure 6:**
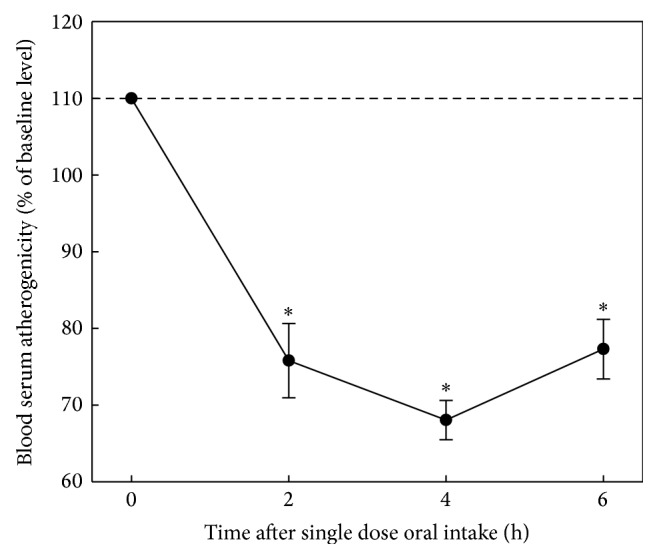
Antiatherosclerotic effect of garlic powder in the* ex vivo* model. The study involved 8 volunteers (6 males, 2 females, mean age 53 ± 5 years) whose blood serum induced 1.3–2.7-fold increase in cholesterol content of cells cultured from unaffected human aortic intima (the average level of serum atherogenicity was 164 ± 9%). Intracellular cholesterol in control cultures was 39.0 ± 4.2 mg/mg cell protein. Baseline serum atherogenicity was taken as 100%. The average values of changes of serum atherogenicity with indication of standard errors are presented. ^∗^Significant decrease of serum atherogenicity, *p* < 0.05.

Wheat seedlings have been found to possess a prolonged and pronounced antiatherosclerotic effect in* ex vivo *model; the effect was manifested in lowering of serum atherogenicity after a single dose of 300 mg preparation (Figure 4). It should be noted that 4 hours after administration of the preparation atherogenicity was completely eliminated. Results of the evaluation of antiatherosclerotic effect of dry beet juice in* ex vivo* model after single dose capsuled preparation (300 mg) are presented in Figure 5. Dry beet juice possessed moderate but prolonged antiatherosclerotic effect in the* ex vivo* model. Garlic powder possessed a pronounced and prolonged antiatherosclerotic effect in the* ex vivo* model [27]; the effect was manifested in lowering of serum atherogenicity after a single dose of 300 mg preparation; 4 hours after administration atherogenicity was completely eliminated (Figure 6). In addition to the above results antiatherosclerotic effects of other natural products have also been revealed. Integral estimation data of antiatherogenic effects of the tested natural products is presented in Table 3. According to the data presented in Table 3, wheat seedlings and garlic powder possess the most pronounced antiatherosclerotic effects in the* ex vivo* model. After a single 300 mg dose they reduced atherogenicity serum 3-fold and the biological effect was observed for 6 hours. However, dynamics of atherogenicity reducing was more pronounced in garlic powder.

To develop antiatherosclerotic therapy which would be based on a natural product, it is essential to determine the effective dose, adequate regime, and course of treatment. For this purpose, patients whose serum possessed atherogenic potential took natural product and their blood was collected after 2 and 4 hours. Patient's serum was incubated with cultured subendothelial cells isolated from unaffected human aortic intima and then intracellular cholesterol was determined. Dose-dependent effect was revealed by comparing the efficacy of two doses. Efficacy of each dose was assessed by the analysis of at least six different sera obtained from patients. Thus, it was found that the garlic powder has antiatherosclerotic effect in a dose range of 50–300 mg and half-maximal effect was achieved at a dose of 100 mg and the maximal effect was achieved at a dose of 150 mg [27]. Thus, the data obtained in experiments utilizing the* ex vivo* model allowed to conclude that natural products including botanicals may be regarded as potential drug substances for the development of direct antiatherosclerotic therapy. Eventually, several preparations that have been registered as dietary supplements have been developed. Clinical studies were conducted to evaluate the antiatherosclerotic efficacy of the three preparations.

## 6. Clinical Studies

**Figure 7:**
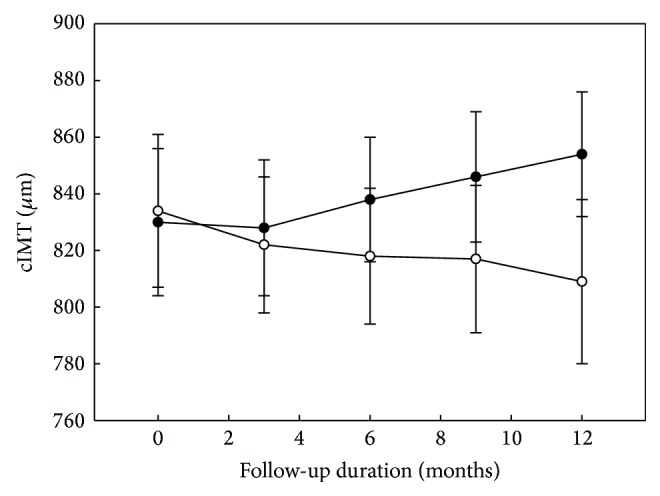
The dynamics of cIMT in open-label pilot study on antiatherosclerotic effects of garlic-based drug Allicor. Open circles, Allicor recipients; solid circles, control subjects. The data are presented in the terms of means and S.E.M.

In an open-label prospective pilot study performed in 28 apparently healthy men aged 46–58 (mean age 52.0, SD = 9.0) the effect of garlic-based dietary supplement (Allicor, INAT-Farma, Russia) on carotid intima-media thickness (cIMT) was estimated. The study participants were normolipidemic or mildly hyperlipidemic and had no clinical signs of coronary heart disease (CHD). Diffused intimal thickening without elevated atherosclerotic lesions was diagnosed by ultrasound B-mode examination of common carotid arteries by the method described elsewhere [28]; the cut-off cIMT value of 0.7 mm in the distal segment of at least one common carotid artery was taken for diagnostics of diffused intimal thickening. Study participants had no chronic diseases requiring continuous use of vasoactive drugs, diuretics, and lipid-lowering or antidiabetic drugs. The mean cIMT value at the baseline was 0.832 ± 0.024 mm. Study participants were divided into 2 groups: 16 of them received 600 mg Allicor daily, and 12 were in the control group. Interview and ultrasound examination of the carotid arteries were held every 3 months, and the total duration of follow-up was 12 months. During the follow-up, no adverse effects were observed, and tolerability was good. The dynamics of cIMT changes are shown in Figure 7. There were no statistically significant changes in cIMT, and by the end of 12-month follow-up the two groups did not differ in cIMT. However, regression analysis revealed a significant difference between the trends in cIMT dynamics (*p* < 0.05). The control group showed a tendency to cIMT increase, which was significantly different from that of null hypothesis of no change (*F*-test, 31.72, *p* = 0.011). In the Allicor-treated group, the tendency to cIMT decrease was revealed, which was also significantly different from that of null hypothesis (*F*-test, 28.81, *p* = 0.013).

The obtained results indicated that the therapy with Allicor might potentially stop the development and induce the regression of subclinical atherosclerosis; the statistical power of this pilot study was insufficient to avoid type 2 error. Therefore, the results of this pilot study allowed us to design the further prospective clinical study with the assessment of a number of clinical and biochemical parameters associated with atherogenesis and the risk of atherosclerosis, including the assessment of the dynamics of serum atherogenicity. It was necessary to increase the number of study participants to achieve statistical power >80%, standardize inclusion and exclusion criteria, use placebo control, and increase the duration of follow-up.

This double-masked placebo-controlled clinical study was designed to estimate the effect of time-released garlic powder tablets Allicor on the progression of cIMT in 211 asymptomatic men aged 40–74. The primary outcome was the rate of progression of subclinical atherosclerosis, estimated by B-mode ultrasonography as the increase in cIMT (Clinicaltrials.gov identifier NCT01734707). By the end of the first 12 months of follow-up, in Allicor-treated group the decrease of cIMT by 0.028 ± 0.008 mm was observed, whereas in placebo group there was a moderate progression at the rate of 0.014 ± 0.009 mm per 12 months (*p* = 0.002 for the difference). Serum atherogenicity (the ability of serum to induce cholesterol accumulation in cultured cells) was lowered in Allicor-treated patients by 45% from the baseline level, on an average. In the placebo group, serum atherogenic potential did not change significantly. These results demonstrated that long-term treatment with Allicor provides a direct antiatherosclerotic effect on subclinical carotid atherosclerosis, and this effect may be due to serum atherogenicity inhibition [7].

**Figure 8:**
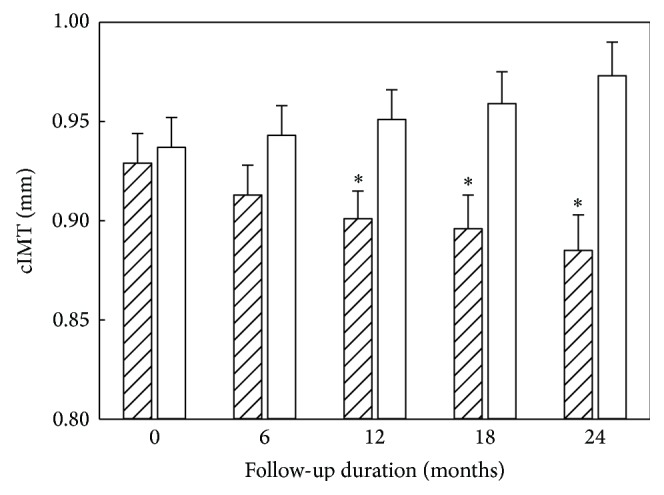
The dynamics of cIMT in double-masked placebo-controlled study on antiatherosclerotic effects of garlic-based drug Allicor. Hatched bars, Allicor recipients; open bars, placebo recipients. The data are presented in terms of means and S.E.M. ^∗^Significant difference between groups, *p* < 0.05.

**Figure 9:**
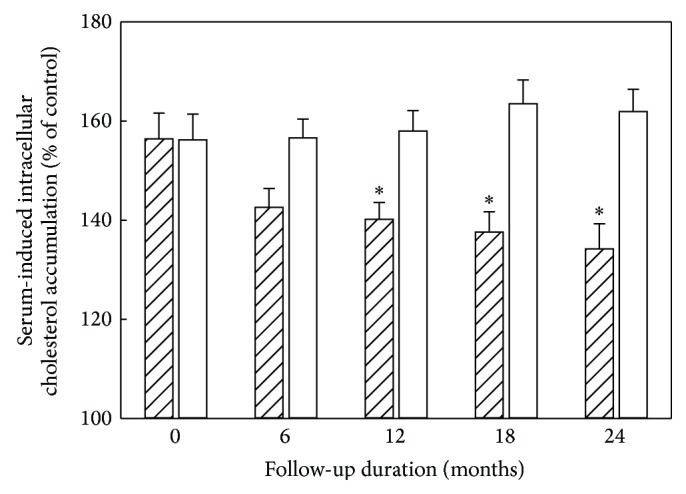
The dynamics of serum atherogenicity in double-masked placebo-controlled study on antiatherosclerotic effects of garlic-based drug Allicor. Hatched bars, Allicor recipients; open bars, placebo recipients. The data are presented in terms of means and S.E.M. ^∗^Significant difference between groups, *p* < 0.05.

By the end of 24-month follow-up, 196 evaluable study participants remained, since 15 discontinued their participation. The mean rate of cIMT decreases in Allicor-treated group accounted for 0.022 ± 0.007 mm per year, which was significantly different (*p* = 0.002) from the placebo group, in which there was a moderate but statistically significant progression of 0.015 ± 0.008 mm at the overall mean baseline cIMT of 0.931 ± 0.009 mm [7]. Within Allicor-treated group, cIMT significant reduction was observed in 47.3% study participants versus 30.1% in placebo group (*p* < 0.05). The further significant cIMT increase was registered in 32.2% study participants in Allicor-treated group versus 47.3% in the placebo group (*p* < 0.05). At the baseline, serum taken from study participants induced 1.56-fold increase in intracellular cholesterol content in cell culture test, on an average. Serum atherogenicity (the ability of serum to induce cholesterol accumulation in cultured cells) was lowered in Allicor-treated study participants by 30% on an average. In the placebo group, serum atherogenic potential did not change significantly during the study. A significant correlation has been revealed between the changes in blood serum atherogenicity during the study and the changes in intima-media thickness of common carotid arteries (*r* = 0.144; *p* = 0.045). The data on the changes in cIMT and serum atherogenicity are shown in Figures 8 and 9. Thus, the results of the above two-year placebo-controlled double-masked study demonstrated that long-term treatment with garlic-based drug Allicor has a direct antiatherosclerotic effect on subclinical carotid atherosclerosis, and this effect may be attributed to serum atherogenicity inhibition [7].

**Figure 10:**
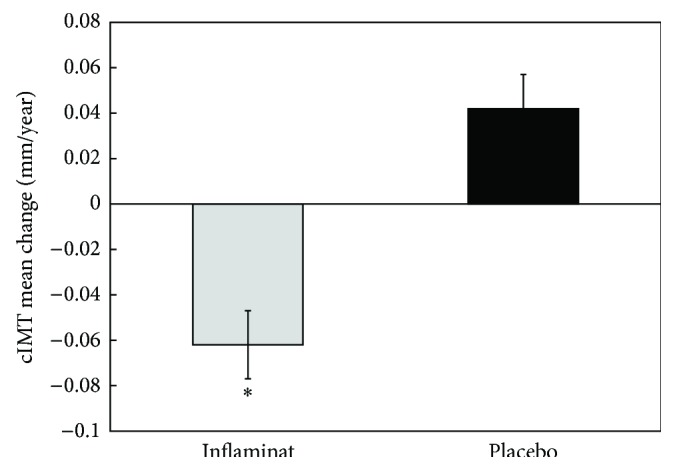
The changes of cIMT in double-masked placebo-controlled study on antiatherosclerotic effects of Inflaminat. The data are presented in terms of means and S.D. ^∗^Significant difference between groups, *p* < 0.05. Adapted from [37], with permission from Bentham Science Publisher.

Atherosclerosis is regarded as a pathological process with the elements of local aseptic inflammation, while inflammatory cytokines play a role at every stage of atherosclerosis development [29, 30]. Therefore, the drugs possessing systemic anti-inflammatory action may be effective for the prevention of atherosclerosis. It has been demonstrated that several natural compounds possess not only anti-inflammatory effect, but also antiatherogenic one: among them are calendula, elder, and violet [31]. On the basis of combination of these three herbs, the dietary supplement has been developed (Inflaminat, INAT-Farma, Russia) [31]. The pilot placebo-controlled double-blinded study has been performed to investigate the effect of Inflaminat on cIMT dynamics in 67 asymptomatic men (Clinicaltrials.gov identifier NCT01743404) [31]. The protocol of the study was similar to that reported above; the duration of follow-up accounted for 12 months. It has been demonstrated that Inflaminat induced cIMT regression in subclinical atherosclerosis, and cIMT changes were statistically significant compared to the baseline as placebo group (Figure 10). Thus, Inflaminat has anti-inflammatory and antiatherosclerotic effects on cellular level revealed in cell culture and induces regression of subclinical atherosclerosis in asymptomatic men.

We screened several natural phytoestrogen-rich components for their antiatherogenic activity using* in vitro* and* ex vivo* test systems [31, 32]. The most promising of these compounds were garlic powder, extract of grape seeds, green tea leafs, and hop cones; all of them produced a significant antiatherogenic effect. On the basis of their combination, isoflavonoid-rich dietary supplement was developed (Karinat, INAT-Farma, Russia). This combination produces the most efficient antiatherogenic effect in cell culture models and is characterized by improved phytoestrogen profile, providing additional amounts of biologically active polyphenols including resveratrol, genistein, and daidzein that are claimed to produce beneficial effects on atherosclerosis development. A randomized double-blinded placebo-controlled pilot clinical study on atherosclerotic effect of Karinat was performed in 157 asymptomatic postmenopausal women to understand the risks and benefits of phytoestrogen therapy in relation to atherosclerosis progression (Clinicaltrials.gov identifier NCT01742000) [31]. The primary endpoint was the annual rate of changes in cIMT. The protocol of the study was similar to that reported above; the duration of follow-up accounted for 12 months.

**Figure 11:**
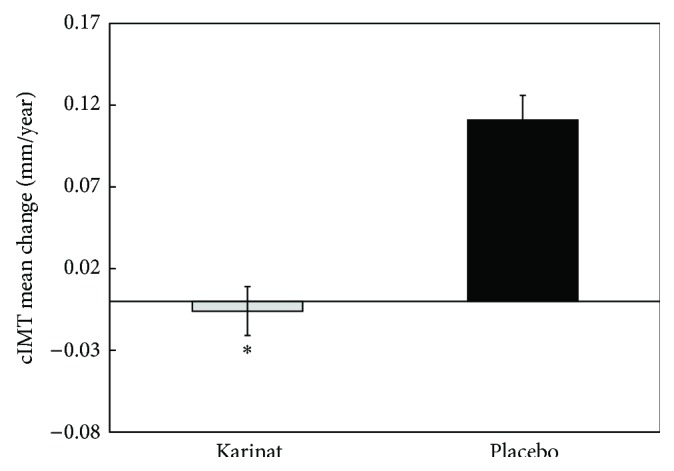
The changes of cIMT in double-masked placebo-controlled study on antiatherosclerotic effects of Karinat. The data are presented in terms of means and S.D. ^∗^Significant difference between groups, *p* < 0.05. Adapted from [37], with permission from Bentham Science Publisher.

In the placebo group, an increase in the mean cIMT of more than 100 *μ*m per year was observed. Thus, the rate of cIMT progression in postmenopausal women was high, as it accounted for 13% per year and growth of atherosclerotic plaques of 40% per year. On the opposite side, in Karinat recipients the mean cIMT did not change; there was statistically insignificant increase of 6 *μ*m per year, that is, less than 1% (Figure 11). In this clinical study, the results of quantitative measurements of the degree of subclinical atherosclerosis in the dynamics have shown that the use of phytoestrogen complex in postmenopausal women almost completely suppresses the formation of new atherosclerotic lesions [31].

## 7. Conclusions

A discovery of the phenomenon of serum atherogenicity significantly promoted the development of simple cell-based models for testing of different types of natural products and drugs with respect to their antiatherosclerotic effects. This approach allowed the development of so called “direct” antiatherosclerotic therapy, the effect of which is realized at the level of the arterial wall, predominantly, via the inhibition of intracellular lipid deposition. To be of potential benefit in patients with established atherosclerosis, a drug should produce regression or slow the progression of atherosclerosis. Further examination of antiatherogenic and antiatherosclerotic effects of various natural products would help the development of novel cardiovascular drugs, possessing mechanistic mode of action on the processes of atherogenesis.

No doubt, there are commonly accepted understanding and appreciation of the fact that any experimental model, including any cell culture model, has some limitations [33–39]. The problem of limitations of experimental models, in particular in atherosclerosis research, has been well highlighted and discussed in a number of comprehensive reviews [36–42]. Despite the existence of unavoidable limitations of any model approach, including the cell culture model approach, the development and principles of which are highlighted in the present review, the data obtained during the clinical trials strongly support a point of view that arterial wall cell models offer a suitable instrument for analysis of effects of drugs and that the discovery of antiatherosclerotic activity of natural products offers great opportunities for the prevention and treatment of atherosclerotic disease, reducing cardiovascular morbidity and mortality.

## Figures and Tables

**Table 1:** Proportion of cell types (identified by cell-defining marker) in primary culture of cells isolated from human aortic subendothelial intima.

Smooth muscle *α*-actin^+^	3G5^+^	2А7^+^	CD45^+^	CD68^+^
89.6 ± 6.7%	45.8 ± 10.9%	24.1 ± 9.9%	3.6 ± 0.4%	5.2 ± 1.3%

**Table 2:** Substances tested with *in vitro* cellular model^∗^.

Substance	
Antiatherosclerotic	
Cyclic AMP	
Prostacyclin	
Prostaglandin E_2_	
Artificial high-density lipoprotein (HDL)	
Antioxidants	
Calcium antagonists	
Trapidil and its derivatives	
Lipoxygenase inhibitors	
Lipostabil	
Mushroom extracts	
Proatherogenic	
Beta-blockers	
Thromboxane А_2_	
Phenothiazine	
Indifferent	
Nitrates	
Cholestyramine	
Sulfonylureas	

^∗^Adapted from [31], with permission from Bentham Science Publisher.

**Table 3:** Integral estimation of antiatherogenic actions of natural products^∗^.

Botanical and its source	The mean efficiency of atherogenic reduction, %	Maximum effect, %
*Spirulina platensis* powder	50.7%	61
Onion (*Allium cepa*) bulb powder	21.4%	28
Beet (*Beta vulgaris*) juice powder	30.7%	40
Wheat (*Triticum vulgaris*) seedlings powder	70.0%	100
Licorice (*Glycyrrhiza glabra*) root powder	54.6%	32
*Salsola collina* leaf powder	10.9%	28
Garlic (*Allium sativum*) bulbs powder	76.6%	100
Pine (*Pinus sylvestris*) needles extract	52.1%	62

^∗^The integrated effect was calculated as a mean reduction in serum atherogenicity for 6 hours after a single oral dose.
